# Personalized Medicine in Gastrointestinal Stromal Tumor (GIST): Clinical Implications of the Somatic and Germline DNA Analysis

**DOI:** 10.3390/ijms160715592

**Published:** 2015-07-09

**Authors:** Gloria Ravegnini, Margherita Nannini, Giulia Sammarini, Annalisa Astolfi, Guido Biasco, Maria A. Pantaleo, Patrizia Hrelia, Sabrina Angelini

**Affiliations:** 1 Department of Pharmacy and Biotechnology, University of Bologna, via Irnerio 48, 40126 Bologna, Italy; E-Mails: gloria.ravegnini2@unibo.it (G.R.); giulia.sammarini@studio.unibo.it (G.S.); patrizia.hrelia@unibo.it (P.H.); 2 Department of Specialized, Experimental and Diagnostic Medicine, Sant’Orsola-Malpighi Hospital, University of Bologna, via Massarenti 9, 40138 Bologna, Italy; E-Mails: margherita.nannini@unibo.it (M.N.); guido.biasco@unibo.it (G.B.); maria.pantaleo@unibo.it (M.A.P.); 3 “Giorgio Prodi” Cancer Research Center, University of Bologna, via Massarenti 11, 40126 Bologna, Italy; E-Mail: annalisa.astolfi@unibo.it

**Keywords:** GIST, KIT/PDGFRA mutant GIST, WT-GIST, personalized therapy, biomarkers, drug resistance, polymorphisms

## Abstract

Gastrointestinal stromal tumors (GIST) are the most common mesenchymal tumors of the gastrointestinal tract. They are characterized by gain of function mutations in KIT or PDGFRA tyrosine kinase receptors, with their consequent constitutive activation. The gold standard therapy is imatinib that offers a good and stable response for approximately 18–36 months. However, resistance is very common and it is vital to identify new biomarkers. Up until now, there have been two main approaches with focus to characterize novel targets. On the one hand, the focus is on the tumor genome, as the final clinical outcome depends mainly from the cancer specific mutations/alterations patterns. However, the germline DNA is important as well, and it is inconceivable to think the patients response to the drug is not related to it. Therefore the aim of this review is to outline the state of the art of the personalized medicine in GIST taking into account both the tumor DNA (somatic) and the patient DNA (germline).

## 1. Introduction

Gastrointestinal stromal tumors (GIST) are the most common mesenchymal tumors of the gastrointestinal tract and are worldwide considered a paradigm of molecular biology in solid tumors [1]. Everything has begun with the introduction of tyrosine-kinase inhibitors (TKI) that positively affected the long-term prognosis of GIST patients, tremendously modifying the natural history of this rare disease [2,3,4]. Imatinib was the first TKI introduced in GIST management in 2000, and still remains the only approved first line treatment, while sunitinib and regorafenib represent the second and third line treatment, respectively [2,3].

The GIST paradigm has been proven to be more complex than expected, due to the evidence of a molecular heterogeneity within all GIST tumors, and the identification of different subgroups frequently characterized by a peculiar genotype-phenotype [5]. With the discovery of the common and mutually exclusive KIT or platelet-derived growth factor receptor alpha (PDGFRA) gain-of-function mutations, which occur in about 70%–80% and 7% of cases respectively, deeper insights on GIST biology have been progressively gained. Specifically, with the application of high throughput technologies into basic and translational research, we experienced the identification of a wide spectrum of other genomic alterations [6,7,8,9,10]. The biological role of most of these additional events in GIST pathogenesis and development remains undefined. However, it is known that acquisition of secondary resistance to TKIs’ frequently shows substantial heterogeneity within and between metastases from individual patients [11].

Tumor handling is, and has been for a long time, one of the most difficult issues. In the era of personalized medicine, clinicians are faced with many intriguing doubts regarding the choice of the adequate drug administered at the correct dosage, for a certain patient. Unfortunately, regarding GIST patients, clinicians do not have a real choice since only TKIs are approved for GIST treatment, even for patients without TK-gain of function mutations. In this context, the rapid progress of high-throughput genome sequencing, applied to molecular diagnosis, has the potential to drive the development and approval of new therapeutic options for GIST patients resistant to TKIs or with mutations in genes other than TK [12]. What is known to be sure is that two main players are involved in the final clinical outcome: the somatic DNA and the germline DNA [12]. The somatic genome represents the specific tumor DNA and includes all the mutations and alterations strictly associated with the cancer. The germline DNA represents the patient’s genome. These two genomes together are relevant in cancer treatment, dictating response and toxicity. In particular the somatic DNA influences the tumor behavior and aggressiveness, and mainly it determines the responsiveness to the treatment; germline DNA primarily influences drug exposure (how the body handles) and toxicity (how the body reacts) [12].

In this view, scientists and clinicians are obligated to take into account that both DNAs supply specific biomarkers which together affect the patient’s drug response. Therefore, from the *era of targeted therapies* we are moving towards the *era of personalized therapies*, which should take into account all genomic variables, both the tumors and the patients, making GIST a more actual model in the molecular biology of solid tumors. In light of these considerations, the integrated study of the somatic genome and the germline DNA certainly represents a major step towards the translation of personalized therapy into clinical practice.

The aim of this review is to outline the state of the art of the personalized medicine in GIST considering two sides of the same coin: on the one hand the tumor DNA, with its mutations pattern that strictly typify the cancer, and on the other hand the patient DNA, that could contribute to the clinical outcome as well.

## 2. Pharmacogenetic Approaches in GIST: State of the Art

As mentioned above, approximately 80% of GIST harbors KIT/PDGFRA mutations, however, unfortunately, there are not inherited genetic factors. Epidemiological studies have raised the possibility that risk factors, e.g., dioxin and radiation exposure, may be linked to sarcomas [13]. In regard to these considerations, O’Brien and colleagues analyzed 208 variants in 39 candidate genes related to DNA repair and dioxin metabolism and imatinib response in 279 GIST enrolled in a clinical trial for neoadjuvant imatinib. In particular, they found that polymorphisms in CYP1B1 (rs2855658 and rs1056836) were strongly associated with the presence of KIT exon 11 codon 557-8 deletions (*p* = 0.002 and *p* = 0.004, respectively). Moreover, they found other potential risk variants such as RAD23B, ERCC2 and GSTM1, highlighting the hypothesis of an environmental related origin of GIST [13]. Up until now, no additional studies have been performed to confirm these findings, and this represents the only study, conducted through the high throughput screening platform (Illumina), related to germline variants in GIST.

After imatinib introduction in therapy, GIST patients’ prognosis and survival improved significantly [6,14]. However, despite the excellent results, it is common that patients initially responding well to imatinib, develop progression and acquired resistance through different mechanisms [6,14]. In this view, the major efforts of researchers have focused on identifying the driver mechanisms of acquired resistance as well as novel potential biomarkers for GIST treatment. Though it is likely that in drug response the significance of the tumor DNA weighs more than the germinal one, the genetic code of the patient still remains relevant. Indeed, it is well known that any drug, starting from its intake, undergoes a specific pharmacokinetics itinerary, and a growing body of literature ascribes to this itinerary a role in drug efficacy and side effects [15].

**Figure 1:**
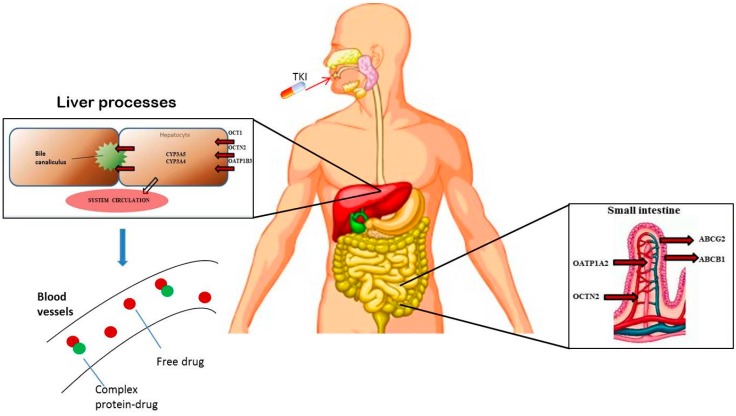
The main players in imatinib pharmacokinetics.

Figure 1 shows the main actors taking part in the resulting imatinib bioavailability; imatinib is almost completely absorbed (~97%) [16], and it is widely metabolized in the liver by the cytochrome P450 isoforms 3A4 and 3A5, while proteins as OCT1, OCTN, OATP, ABCB1 and ABCG2 are transporters affecting its efflux and uptake. As a result, it seems obvious that polymorphisms in genes coding these metabolizing and transporter enzymes could make the differences in the resulting proteins. In a previous pharmacogenetic study evaluating chronic myeloid leukemia (CML) patients undergoing imatinib therapy, an association between imatinib transporter genotype and imatinib response has been found [17]. On the basis of these findings, in 2013, Angelini and colleagues conducted the first pharmacogenetic study on GIST patients undergoing imatinib therapy. Through a multiple candidate gene approach, they analyzed a panel of 31 single nucleotide variants in nine transporters genes and two metabolizing genes in 54 GIST patients treated with imatinib. They found three polymorphisms, one in SLC22A4 (rs105152) and two in SLC22A5 (rs263136, and rs231372)—coding for OCTN1 and OCTN2 transporters respectively—associated with a significant improvement in the time to progression (TTP) [18]. Interestingly, this study confirmed a previous finding in CML patients, highlighting the involvement of the OCTN1 polymorphisms (rs105152) in imatinib response [17].

More recently, Koo *et al.* reported a polymorphism in ABCG2 (rs2231142) associated with five-year progression-free survival (PFS) in 209 GIST treated with imatinib 400 mg/daily; in particular, carriers of the AA genotype had a longer PFS compared with GG or AG carriers [19]. This represents an intriguing finding as the same polymorphism has been analyzed by Angelini *et al.* showing no significant correlation. The discrepancy might be due to the small GIST population analyzed by Angelini *et al.* (54 *vs* 209 GIST patients), with a consequent low statistical power, which might not have revealed the significance [17,19]. To the best of our knowledge, there are no additional studies in GIST reporting on polymorphisms in imatinib transporters and metabolizing genes.

In the last years, growing evidence of a strict link between aberrant methylation profile and cancer, as well as neurodegenerative diseases, have been reported [20,21]. In particular, a recent finding suggests the DNA methylation pattern may be associated with aggressive clinical behavior and unfavorable prognosis in GIST [22]. Based on these findings, and considering that the genes coding for enzymes involved in the folate pathway could impact the methylation processes, Angelini *et al.* evaluated 13 common polymorphisms in eight of the main folate-related genes, in 60 GIST patients and 153 healthy controls. The data highlighted a decreased risk of GIST associated with a six bp deletion in the thymidylate synthase (TS) gene (formerly rs34489327, delTInsTTAAAG), and a reduced TTP associated with the wild-type allele in the reduced folate carrier (RFC) [23]. All these results, taken together, suggest the importance of germline genetic variations in genes involved in key pathways, including drug uptake and efflux, and in the accurate regulation of the balance in methylation rate.

The importance of the patient’s DNA is also supported by new evidence reinforcing the role of plasma level regulation in assessing the response rate. In 2010, for the first time, Yoo *et al.* examined imatinib plasma trough levels (*C*_min_) in GIST patients treated with imatinib 400 mg/daily [24]. Among all patients’ and tumor characteristics, the most interesting finding is that major gastrectomy was associated with lower imatinib *C*_min_ when compared to a more conservative gastric resection. This observation suggests that monitoring imatinib *C*_min_ might be particularly important for optimal treatment in patients who have undergone major gastrectomy, as imatinib 400 mg/daily might be insufficient to maintain an optimal blood level, and can be responsible of a poor PFS. However, from this study it is difficult to draw a definitive conclusion on the clinical utility of plasma trough levels monitoring, considering that data on imatinib response were not taken into account [24]. Two years later, Eechoute and colleagues carried out the first prospective pharmacokinetic study in 50 GIST patients, revealing a substantial decrease of approximately 30% in imatinib exposure after long-term treatment, in part due to reduced absorption. According to these results, the authors, in order to get clinical benefits, suggest a different approach that, besides tumor biology, considers also patient characteristics, in particular time point-specific imatinib trough level determination [25]. The same approach has been recommended by Yoon *et al.* that described two case reports with advanced GIST for which imatinib-related toxicities were successfully managed through dose modifications via imatinib plasma level testing [26]. Taken together, these findings indicated that a fine dose adjustment, guided by imatinib plasma level measurement, could prevent over-exposure and the resulting toxicity without affecting its efficacy [25,26]. An important consideration regarding the clinical utility of plasma trough levels monitoring is that optimal threshold value of imatinib *C*_min_ has yet to be determined in GIST patients. Furthermore, even if treatment failure or toxicities could occur with an inappropriate dosing, and imatinib trough levels monitoring could in turn prevent inappropriate dosing, this has not been sufficiently investigated in clinical practice. However, an interesting scenario that has emerged from these studies is that inter-individual differences could impact the resulting drug clearance or the amount of protein bound drug. Unfortunately, to the best of our knowledge, there are no studies in GIST patients evaluating the influence of polymorphisms in imatinib clearance. Interestingly, stimulating information comes from CML patients undergoing imatinib treatment. In particular, Di Paolo *et al.* reported a correlation between the hOCT1 polymorphisms (c.480C>G, rs683369) and drug clearance in 60 CML patients treated with imatinib [27]. In this study, patients carrying at least one polymorphic G-allele had a significantly lower drug clearance than CC carriers. This intriguing finding should stimulate further research in GIST patients and, if validated, it could be useful in choosing the most effective dose with the as low toxicity as possible.

**Table 1:** Summary of the pharmaocogenetics studies.

Authors	Year	*#n* of the Evaluated SNPs and Pathway	Gene/Reference Sequence (rs) *	*#n* of Cases	Aim	Significant SNPs
O’Brien *et al* [13].	2013	208 SNVs in 39 candidate genes related to DNA repair and dioxin metabolism or response	*CYP1A2*, *CYP1B1*, *HIF1A*, *NQO1*, *G6PC/G6PT*, *ADH1A*, *ADH1B*, *ADH1C*, *ALDH18A1*, *ALDH1A1*, *ALDH1A2*, *ALDH1A3*, *ALDH1B1*, *ALDH1L1*, *ALDH1L2*, *ALDH2*, *CYP2B6*, *CYP2C8*, *CYP2C9*, *CYP2D6*, *CYP2E1*, *CYP3A4*, *GSTM1*, *GSTT1*, *GSTP1*, *HNF4A*, *NAT2*, *NFE2L2*, *NOS2A*, *PTGS2/COX2*, SULT1A1, TP53, MDM2.	279 GIST from a clinical trial of adjuvant imatinib mesylate	To test the association between germline SNVs and somatic mutations and to evaluate the hypothesis of environmental related origin for GIST	CYP1B1 rs2855658 and rs1056836 were associated with KIT exon 11 codon 557-8 del; ERCC2 rs50871 was associated with WT GIST; ERCC2 rs50871 was associated with KIT exon 11 insertion (no codon 557-8); GSTM1 deletion was associated with KIT exon 11 codon 557-8 del; RAD23B rs1805329 and rs7041137 were associated with other KIT mutations (none in exon 11)
Angelini *et al* [18].	2013	27 SNVs in 9 transporters genes; 4 SNVs in 4 metabolizing genes	SLC22A1 (rs12208357, rs683369, rs4646277, rs4646278, rs2282143, rs72552763); SLC22A4 (rs1050152); SLC22A5 (rs2631367, rs2631370, rs2631372); SLCO1A2 (rs11568563); SLCO1B3 (rs4149157, rs4149158, rs4149117, rs7311358); ABCA3 (rs323040, rs4146825); ABCB1 (rs10245483, rs3213619, rs1128501, rs1128503, rs60023214, rs2032582); ABCC4 (rs3765534, rs9561765); ABCG2 (rs2231137, rs2231142); CYP3A4 (rs2740574, rs28371759); CYP3A5 (rs776746, rs28365083).	54 GIST patients receiving imatinib 400 mg	To evaluate the correlation among SNPs and clinical outcome	TTP improved by C allele in SLC22A4 (rs1050152; *p =* 0.013), and by G alleles in SLC22A5 (s2631367; *p =* 0.042) and (rs2631372; *p =* 0.045)
Koo *et al* [19]*.*	2015	5 SNVs in 2 transporters genes; 1 SNVs in 1 metabolizing genes	ABCB1 (rs1128503, rs1045642, rs2032582); ABCG2 (rs2231137, rs2231142); CYP3A5 (rs776746).	209 GIST patients receiving imatinib 400 mg	To evaluate the correlation among SNPs and clinical outcome	The 5-year PFS rate in patients with the AA variant of ABCG2 rs2032582 was superior compared with patients with CC/CA genotypes (*p* = 0.047)
Angelini *et al* [23]*.*	2015	13 SNVs in 8 folate pathway genes	RFC (rs1051266); FOLR (rs2071010); DHFR (rs70991108); TS (rs45445694, rs34489327); SHMT (rs1979277); MTHFR (rs1801131, rs1801133); MTR (rs1805087); MTRR (rs10380).	60 GIST patients receiving imatinib 400 mg and 153 controls	To evaluate the correlation among SNPs and clinical outcome	In 54 patients, presence of WT allele in RFC rs1051266, (AA/AG) was associated with reduced TTP (*p =* 0.028)
Rutkowski *et al* [28].	2012	6 SNVs in 2 VEGF pathway genes	VEGFA (rs699947, rs3025039,rs2010963, rs833061); VEGFR2 (1531289, rs1870377).	39 GIST patients receiving sunitinib 2nd line treatment 50 mg	To evaluate the correlation among SNPs and adverse reactions or toxicity	Presence C-allele in VEGFA rs833061 and the T-allele in rs3025039 were associated with higher risk of hypothyroidism (*p* = 0.041 and *p* = 0.015, respectively)

* The polymorphisms analyzed by multiple studies are highlighted in red.

To date, most of the pharmacogenetics literature is centered on imatinib while the potential associations with sunitinib or regorafenib have not been taken into account. The only exception is represented by the work of Rutkowski and colleagues who described the outcome and potential predictive factors in GIST patients treated with sunitinib after imatinib failure [28]. In this study, six polymorphisms in genes belonging to the vascular endothelial growth factor (VEGF) pathway were evaluated. Sunitinib is a multitargeted agent, an inhibitor of tyrosine kinases, including KIT, PDGFRA/B and the VEGFR receptors −1 and −2. Therefore, as sunitinib acts through the VEGF signaling cascade, authors investigated the influence of VEGFA and VEGFR2 polymorphisms on sunitinib toxicity, highlighting a likely link between sunitinib-induced hypothyroidism [28]. Regrettably, given the small population size of 39 cases, the authors did not evaluate any possible association with the clinical outcome and at the moment no data are available. Table 1 summarizes the studies and shows the polymorphisms identified as involved in the pharmacogenetics of GIST. To the best of our knowledge, no other pharmacogenetic studies have been conducted on sunitinib or regorafenib. However, considering the similar pharmacokinetics of the two drugs with the cytochrome P450 isoforms 3A4 and 3A5 as main players in their metabolisms, and proteins ABCB1 and ABCG2 as transporters, it is plausible that polymorphisms in these genes may also affect their clinical utility.

A fascinating part of pharmacogenetics is left for the emerging role of microRNAs as potentially responsible for clinical outcome variability. MicroRNAs are a class of 18–24 nucleotides long noncoding RNAs, playing an important role in significant biological processes, such as differentiation, proliferation and apoptosis [29]. MicroRNAs alteration represents epigenetic modifications that are emerging as having a relevant role in GIST biology, including disease development and progression, clinical outcome and drug resistance [30]. Unfortunately, as highlighted by Nannini *et al.*, despite the growing evidence of the importance of microRNAs in GIST, there is a small consensus among the microRNA signature between the different studies [31]. However, the majority of them agreed about the involvement in GIST pathogenesis of miR-221 and miR-222, which have been reported by four independent groups as implicated in KIT expression regulation [32,33,34,35]. In particular, it has been demonstrated that their overexpression negatively regulates the TK receptor expression rate and consequently miR-221 and 222 could be realistically developed as therapeutic-targets for GIST management.

## 3. Analysis in Somatic DNA

The detection of somatic mutations from cancer genome sequences is the key to understanding the genetic basis of disease progression, patient survival, therapy response and toxicity. The tumor DNA is the main source of information, which has led over the years to a more detailed characterization of the biological profile of GIST. According to the somatic genome, GIST are divided into KIT/PDGFRA mutant GIST and wild-type (WT) GIST, characterized by deep differences in imatinib response. GIST KIT exon 11 mutants manifest response rate in 80% of cases, KIT exon 9 in 40%, and GIST WT in 14%. PDGFRA mutants show a mild sensitivity to imatinib (66%), with the exception of exon 18 point mutation (D842V), which is totally resistant [10,11,12].

### 3.1. KIT/PDGFRA Mutant GIST

From the beginning, the kinase mutational status has represented the peculiar molecular hallmark of GIST, and has been recognized as the main pathogenic event as well as the best known predictive biomarker of tumor response to TKI [36,37,38,39]. Besides the importance of GIST mutational status in predicting imatinib sensitivity, as described above, the acquisition of secondary mutations in either KIT or PDGFRA represents the most frequent mechanism of imatinib resistance in GIST. The acquisition of additional KIT and PDGFRA mutations, occurs with a median of 24 months and shows a typical heterogeneous distribution within each lesion [40]. However, other molecular events beyond KIT and PDGFRA mutations may play a relevant role in GIST pathogenesis and progression, even if at the moment they remain still unidentified. For example, it has been shown that micro-GIST, defined as GIST smaller than 1 cm, can display KIT/PDGFRA mutations and, at the same time, carry rare and novel mutations, suggesting that, actually, other pathogenic molecular events, besides kinase mutations, could occur [41]. Furthermore, secondary resistance can also occur without the onset of secondary KIT/PDGFRA mutations, suggesting that other driven molecular events can play a relevant role in GIST biology. So, in recent years, with the use of increasingly innovative high throughput technologies, many efforts have been made in order to better characterize the molecular background of each GIST subtype. This, in the long run will lead to the identification of novel targets, thus expanding the armamentarium of GIST therapies available to clinicians. Most of the studies showed that GIST display cytogenetic aberrations, with the highest occurrence of 1p, 13q, 14q, and 15q loss, and 22q loss of heterozygosity. Interestingly, this unstable karyotype typifies mutant GIST, whereas WT GIST do not show genomic imbalances [8,42,43,44]. Integrating the high-resolution genomic copy number analysis with gene expression profiling, some known oncogenes, including KRAS in chromosome 12p amplification, and some tumor suppressors genes, such as KIF1B, PPM1A, and NF2 on chromosome 1p, 14q and 22p deletions, respectively, have been found. Moreover, other tumor suppressor genes, including DAAM1, RTN1 and DACT1 have been restricted to the 14q23.1 region, which represents the genomic segment most frequently altered in mutant GIST [8]. In a further study, it has been shown that the ETS family transcription factor, ETV1, is universally highly expressed in GIST, both at protein and mRNA levels, and at higher levels than any other tumor type, including melanoma and prostate cancer [44]. Additionally ETV1 is required for tumor growth and survival in both imatinib-sensitive (GIST882) and imatinib-resistant (GIST48) cell lines. Furthermore, ETV1 is highly expressed in the subtypes of interstitial cells of Cajal (ICC)—the presumed GIST cell of origin—sensitive to oncogenic KIT mediated transformation, and required for their development into GIST [44]. ETV1 is a master regulator of the ICC lineage, and a recent study in a mouse model of KIT activation and ETV1 ablation has demonstrated that ETV1 is required for GIST initiation and proliferation *in vivo* [45]. Furthermore, a positive feedback circuit involving MAPK signaling, that stabilizes ETV1 protein, which in turn positively regulates KIT expression has been found [45]. Taken together, these results suggest that ETV1 may be considered a new key therapeutic target in GIST, with the potential to revolutionize the first-line treatment of GIST patients [45].

### 3.2. Wild-Type GIST

While the efforts for mutant GIST have been addressed to the discovery of other oncogenic events beyond KIT and PDGFRA, the efforts for KIT/PDGFRA WT GIST have been directed to the identification of the molecular signature of this small subset of GIST. For a long time, WT GIST have been viewed as a unique and less frequent subgroup of GIST, characterized by the lack of known KIT and PDGFRA mutations. Advances in science, culminating with whole genome analysis have shown that KIT/PDGFRA WT GIST should be considered more appropriately as a heterogeneous family of distinct disease entities, with different biological and clinical features [46]. As previously highlighted, WT GIST are characterized by an imatinib response rate of 14%, requiring the identification of novel targets and the development of new therapeutic strategies. About 15% of KIT/PDGFRA WT GIST harbors activating mutation in BRAF or, more rarely, RAS gene [47]. Effective treatments for BRAF-mutant GIST might be the use of a BRAF inhibitor such as dabrafenib, which is active in several BRAF-mutant cancers [48]. To the best of our knowledge, the efficacy of BRAF inhibitors in BRAF-mutant GIST has not been reported, however, there is a case of prolonged antitumor activity in a BRAF-mutant GIST, which suggests the need for additional studies and perhaps a global clinical trial [49].

In addition, KIT/PDGFRA WT GIST may be related to syndromic neurofibromatosis type I (NF1) disease, associated with NF1 protein loss of function due to genomic inactivation of the NF1 gene [50]. Moreover, about 20%–40% of KIT/PDGFRA WT GIST shows a loss of function of the succinate dehydrogenase (SDH) complex, identified with the loss of the subunit B (SDHB) protein expression and referred as SDH-*deficient* GIST or type 2 GIST. All the SDHB immunohistochemical-negative GIST are also characterized by an over expression of the insulin growth factor 1 receptor (IGF1R) [51,52]. With regard to SDH-*deficient* GIST, they display distinctive clinic-pathological features, including predominance in young (<40 years) women, gastric localization, multifocality, mixed epitheliod and spindle cell morphology, diffuse KIT and DOG1 positivity, frequent lymphonode metastases, and an indolent course, although it is often metastatic when diagnosed [53]. In most cases, SDH-*deficient* GIST harbor germline and/or somatic loss-of-function mutations in any of the four SDH subunits (A, B, C, or D; SDH*x*), with the highest occurrence of SDHA mutations [54,55]. More recently, it has been shown that loss of SDHB protein expression, not driven by SDH*x* mutations, might be due to a hypermethylation in the SDHC promoter region [9,56].

Most recently, a small subgroup of KIT/PDGFRA WT GIST, referred as *quadruple* WT GIST, that lack mutations in any of the known KIT exons (8, 9, 11, 13, 14, 17) or PDGFRA exons (12, 14, 18) or RAS pathways, including BRAF (exons 11, 15) and RAS (exons 2, 3), or NF1, and yet retain an intact SDH complex (SDHB IHC positive, and no mutations in SDH*x*) has been identified [57,58]. A whole genome analysis, using massively parallel sequencing and gene expression analysis, has shown that *quadruple* WT GIST have an expression signature extremely different from both KIT/PDGFRA mutated and SDHA mutated GIST, characterized by the overexpression of molecular markers (CALCRL and COL22A1) and of specific oncogenes, including tyrosine and cyclin-dependent kinases (NTRK2 and CDK6), and one member of the ETS-transcription factor family (ERG) [58].

All this evidence together reveals that GIST should be considered as a distinctive set of biological entities, and it would be wise to take into consideration all these genomic variables when selecting the medical treatment. Unfortunately, the high cost associated with genomic analysis, as well as the need of specialized laboratories and tools, and the need of fresh tumor tissue for analysis, restrict the realization and its application in clinical practice. For these reasons, in recent years, the DNA extracted from formalin-fixed, paraffin-embedded specimens and, more recently, the circulating tumor DNA (ctDNA) obtained from a patient’s bloodstream, known as liquid biopsy, have become the most potential and promising source of tumor DNA [59,60]. In particular, the possibility to extend sophisticated analysis with high-throughput technologies on all archived specimens, may allow genomic studies on a wider number of samples, and thereby improve the knowledge of the biological background of all subsets of GIST. Furthermore, in view of a personalized therapy, serial evaluations of ctDNA may offer a dynamic picture of the molecular changes during the course of the disease, in order to identify the early development of heterogeneous resistant clones, allowing optimizing the time to switch the treatment, as well as the choice of the right treatment. All this should lead to the identification of the optimal therapeutic strategy for each individual patient.

## 4. Conclusions

The prognosis for GIST patients has changed enormously over the last decades. In particular, imatinib has radically changed life expectancy of patients with GIST, a previously largely untreatable group of patients. However, imatinib has not proven to be the definitive answer for their management. For those with disease refractory to imatinib, as well as the great majority who develop resistance to imatinib, other TKI, sunitinib and regorafenib, are available. It is clear that, at the moment, TKIs are the only available treatment in GIST, however, with the development of high throughput technologies, as next generation sequencing, and the use of ctDNA, new targets will be available and new targeted therapies will be developed. The increasing number of targeted agents, which hold promise for improving outcomes in patients with GIST, raises interesting questions about the optimal utilization of these agents and the development of pharmacogenetics tests. In the most ideal situation pharmacogenetics will allow oncologists to individualize therapy based on somatic and germline genetic test results. Overall, this can help to improve efficacy, reduce toxicity and predict non-responders in hope that alternative therapy can be chosen or individual dose adjustments can be made. Despite the extensive studies and some promising results, it remains unclear when and how pharmacogenetic testing should be routinely integrated into GIST management.

Very few genotype-driven dose-optimization studies prospectively assessed objective response rate, progression-free survival, overall survival or other measures of efficacy as their primary endpoints, and have been translated into clinical practice. In regard to GIST, it is still an uphill road towards personalized medicine. The evidence is still too sparse to provide a solid relationship between germline variants and imatinib response, largely due to a lack of validated predictive polymorphisms. Furthermore, the way to the identification of translatable pharmacogenetic markers may be complicated by the inability to always take into account the effects of somatic genome, tumor heterogeneity, epigenetic factors, or additional unidentified prognostic factors.
